# An 8-year longitudinal study of long-acting injectable (LAI) antipsychotics. Prescription trends and therapeutic drug monitoring to inform precision dosing

**DOI:** 10.1192/j.eurpsy.2022.482

**Published:** 2022-09-01

**Authors:** D. Piacentino, F. Carpi, G. Giupponi, A. Conca

**Affiliations:** 1 National Institutes of Health, National Institute On Drug Abuse/national Institute On Alcohol Abuse And Alcoholism, Bethesda, United States of America; 2 Central Hospital of Bozen, Department Of Psychiatry, Bozen, Italy

**Keywords:** psychopharmacology, Precision Psychiatry, Long-acting injectable antipsychotics, Therapeutic drug monitoring

## Abstract

**Introduction:**

Despite the widespread use of long-acting injectable (LAI) antipsychotics in schizophrenia and other disorders, there is a lack of longitudinal studies evaluating prescription trends and the usefulness of therapeutic drug monitoring (TDM) to inform dosing. Indeed, LAI prescription varies greatly among different areas of the world and over the years.

**Objectives:**

Assess trends in LAI prescription in 2013-2020 at the Psychiatry Department of Bozen, Italy, and (2) analyze the correlation between dose of prescribed LAIs and blood levels measured via TDM.

**Methods:**

Parametric statistics.

**Results:**

LAIs were administered to 471 patients (x̅ age±SD=47.2±16.3 years; 56.3% men). The pie chart shows LAI treatment duration, i.e., from 1 to 7 consecutive years. The most used LAIs were haloperidol in 2013-2104 (26.5-31.8%) and paliperidone in 2015-2020 (22.5-25.7%). Dose adjustments were rather frequent, whereas the switch between LAI, due to moderate-to-side effects or unsatisfactory improvement of clinical status, was infrequent (41 cases/8 years). LAI interruption for the same reasons or non-compliance was even more infrequent (10 cases), but in 8 cases it happened for opposite reasons, i.e., achievement of patients’ stabilization and good compliance. The Table shows doses and plasma levels of LAIs. Correlations between doses and plasma levels were: haloperidol: r=-0.037, p=0.620; paliperidone: r=0.290; p=0.000; risperidone: r=0.219, p=0.006; fluphenazine r=0.358, p=0.000; aripiprazole: r=-0.051, p=0.610; olanzapine: r=-0.090, p=0.634.

**Figure:**
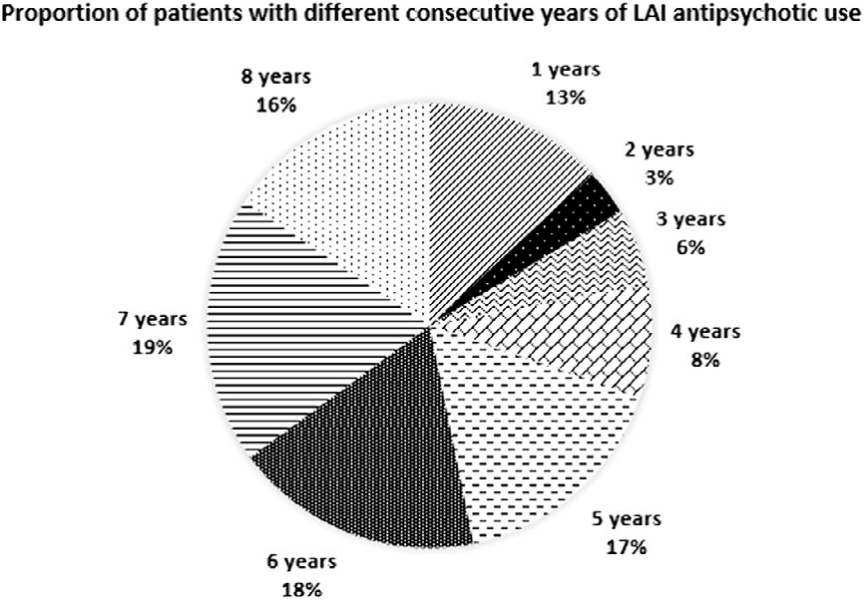

**Figure:**
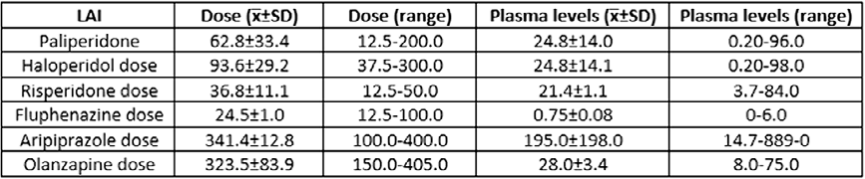

**Conclusions:**

Haloperidol and paliperidone were the most used LAIs. Drug prescription trends and doses were stable over time. A significant positive correlation between dose and plasma level was found for paliperidone, fluphenazine, and aripiprazole.

**Disclosure:**

No significant relationships.

